# Extending the Frequency Range of Surface Plasmon Polariton Mode with Meta-Material

**DOI:** 10.1007/s40820-016-0110-8

**Published:** 2016-09-27

**Authors:** Fengyu Zhou, Fang Liu, Long Xiao, Kaiyu Cui, Xue Feng, Wei Zhang, Yidong Huang

**Affiliations:** Department of Electronic Engineering, Tsinghua National Laboratory for Information Science and Technology, Tsinghua University, Beijing, 100084 People’s Republic of China

**Keywords:** Plasmonics, Surface plasmon polariton, Hyperbolic meta-material, Surface mode

## Abstract

The frequency range that surface plasmon polariton (SPP) mode exists is mainly limited by the metal material. With high permittivity dielectrics above metal surface, the SPP mode at high frequency has extremely large loss or can be cutoff, which limits the potential applications of SPP in the field of optical interconnection, active SPP devices and so on. To extend the frequency range of SPP mode, the surface mode guided by metal/dielectric multilayers meta-material has been studied based on the theory of electromagnetic field. It is demonstrated that surface mode not only could be supported by the meta-material but also extends the frequency to where conventional metal SPP cannot exist. Meanwhile, the characteristics of this surface mode, such as dispersion relation, frequency range, propagation loss and skin depth in meta-material and dielectrics, are also studied. It is indicated that, by varying the structure parameters, the meta-material guided SPP mode presents its advantages and flexibility over traditional metal one.

## Introduction

Surface plasmon polariton (SPP) has been an attractive and extensively studied topic in the scientific community for its various unique features. It has shown promising applications in many fields such as highly integrated optical circuits, high sensitive biological sensing, enhancing light-matter interaction, and so on [1–6].

Although SPP modes can be designed by varying the metal structure, the frequency range and propagation loss still depends on the metal material. For example, on the semi-infinite metal surface, SPP mode cannot exist within the frequency range of $\omega > \omega_{\text{sp}} = \frac{{\omega_{\text{p}} }}{{\sqrt {\varepsilon_{\infty } + \varepsilon_{1} } }}$ [1, 7], where *ω*_p_ is the plasma frequency of metal, *ω*_∞_ is the background permittivity of metal, and *ε*_1_ is the permittivity of semi-infinite dielectric upon the metal. Dielectrics with large permittivity *ε*_1_, such as Si or GaAs which are well used in some functional SPP devices [8, 9], would lead to the decrease of *ε*_sp_ and the cutoff of SPP mode. In some cases, even though SPP mode is not cutoff, the surrounding with large permittivity would result in extremely large propagation loss at certain optical frequency range.

Meta-material at optical frequency can be regarded as a kind of man-made material with properties beyond natural materials [10]. One of its prominent properties is that the equivalent permittivities on different directions differ from each other and can be varied by designing its structure elaborately [10]. This property promises that meta-material may serve for SPP propagating with more flexible performances than conventional metal to deal with the problems mentioned above. As we know, the study of SPP on meta-material is mainly based on abstracted models by assuming the permittivity of the material as fixed indefinite tensors rather than functions of the specific physical structure [2], so that the conclusions are more concerned about the generality instead of pertinence.

In this paper, the surface mode existing on the surface of the meta-material structure composed of alternate layers of metal and dielectric material is studied. The surface mode can be guided by the multilayers structure and represents some unexpected properties. The dispersion relation of the surface mode for multilayers is deduced and the corresponding characteristic parameters are computed. Later analysis indicates that this surface mode originated from the reaction of electromagnetic field and the free electrons oscillation in metal film, and thus it is a kind of SPP mode.

Compared with the conventional pure metal guided SPP, the multilayer-guided SPP has larger mode field distribution and much lower energy loss at the same frequency. More importantly, it is amazing that this structure has the ability to extend the SPP mode to higher frequency surrounding with a high permittivity material, where traditional metal SPP cannot exist. Both the theoretical calculation and the physical explanation are provided to interpret this counterintuitive phenomenon in this paper. Considering the multilayers as an artificial meta-material, it is possible to manipulate the properties of SPP mode by adjusting the structure parameters such as the depth ratio for each layer to meet different actual demands.

## Schematic Structure and Effective Medium Model

**Fig. 1:**
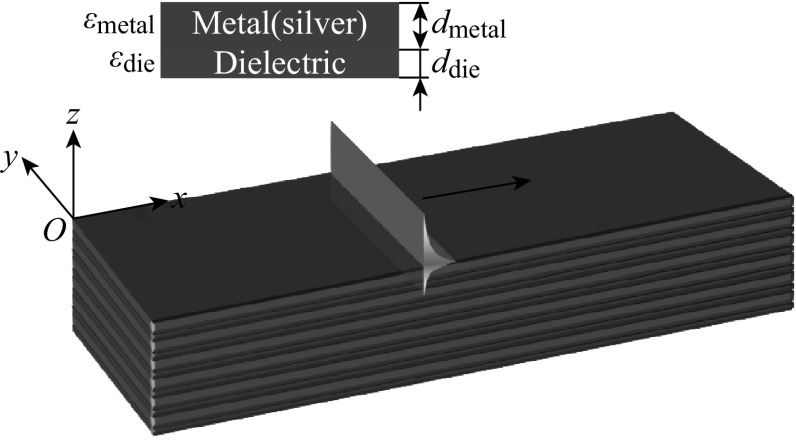
Schematic of metal-dielectric multilayers meta-structure. Here, the gray and brown layers stand for metal (silver) and dielectric, respectively. The multilayers occupy the half-space (*z* < 0), while the other half is isotropic medium *ε*_1_ (*z* > 0)

Figure 1 illustrates the diagram of metal-dielectric multilayer meta-structure, which consists of silver and SiO_2_ layers with thickness ratio *d*_metal_:*d*_die_ = 2:1. The upper space above multilayers (*z* > 0) is filled with isotropic dielectric with permittivity *ε*_1_. The permittivity of SiO_2_ is fixed at *ε*_die_ = *n* ^2^ ≈ 2.4 [11] and the relative permittivity for silver is based on Drude model [12]

1 $$\varepsilon_{\text{metal}} = \varepsilon_{\infty } - \frac{{\omega_{\text{p}}^{2} }}{{\omega^{2} + i\gamma \omega }}$$

where *ε*_∞_ = 5.3, *ω*_p_ = 1.39 × 10^16^ s^−1^, and *γ* = 32 THz [12]. The permittivity of silver might be somewhat different according to different models and measurement methods [13]. Nevertheless, the main conclusion of this paper would not be affected.

When the thickness of both metal and dielectric is around ten nanometers and, therefore, much smaller than the wavelength here, it would be reasonable to use the effective medium theory to describe this structure [14]. According to the effective medium theory, the multilayers are regarded as an anisotropic medium with permittivity *ε*_x_ and *ε*_z_ (the x-axis is along the propagation direction, while the z-axis is perpendicular to the interface). Assuming that the structure is unvarying along the y-axis, this is a two-dimensional problem. By applying the electromagnetic boundary conditions to this model, the approximate value for *ε*_x_ and *ε*_z_ can be expressed as [10, 15, 16]:

2 $$\varepsilon_{x} \approx \frac{{\varepsilon_{\text{die}} d_{\text{die}} + \varepsilon_{\text{metal}} d_{\text{metal}} }}{{d_{\text{die}} + d_{\text{metal}} }}$$

3 $$\varepsilon_{\text{z}} \approx \frac{{d_{\text{die}} + d_{\text{metal}} }}{{\varepsilon_{\text{die}}^{ - 1} d_{\text{die}} + \varepsilon_{\text{metal}}^{ - 1} d_{\text{metal}} }}$$

**Fig. 2:**
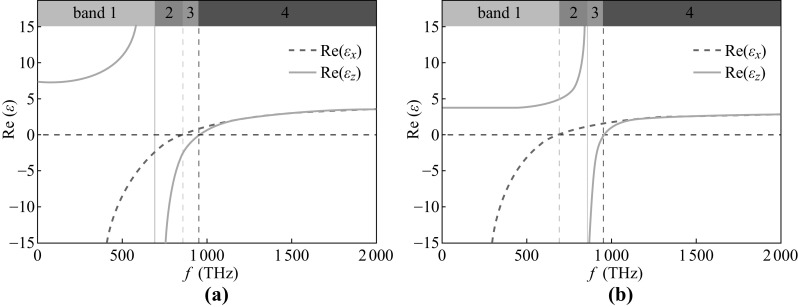
Real parts of *ε*_x_ and *ε*_z_ as a function of frequency. The depth ratio *d*_metal_:*d*_die_ is set at **a** 2:1 and **b** 1:2. According to the signs of *ε*_x_ and *ε*_z_, the frequency can be divided into 4 bands as shown in the figure

According to Eqs. 2 and 3, the *ε*_x_ and *ε*_z_ as functions of the frequency are shown in Fig. 2. In different frequency bands, *ε*_x_ and *ε*_z_ may change their signs and the multilayers exhibit different properties. At low frequency (band 1), the multilayers can be regarded as type II hyperbolic materials [10]. As the frequency goes up, the permittivity in both directions turns negative (band 2). In band 3, the multilayers have the feature of type I hyperbolic materials [10]. If the frequency is high enough, the multilayers become anisotropic dielectric with positive permittivities in both x and z axes (band 4). Here, we mainly focus on band 1 and band 2 to analyze the surface wave of metal-dielectric multilayers. The comparison between Fig. 2a, b indicates that the variation tendency of (*ε*_x_, *ε*_z_) is closely related to *d*_metal_:*d*_die_. For simplicity, we only discuss the situation of *d*_metal_ ≥ *d*_die_, so the permittivity applied in following passages will be based on Fig. 2a.

## The Dispersion Relationship

In analogy to the traditional metal-SPP, it is reasonable to suppose that the surface wave of metal-dielectric multilayers also results from the coupling between electromagnetic field and electronic oscillation [1, 17]. The wave is bounded to the interface of meta-material and propagates along the interface. In the normal direction, the electromagnetic field decays exponentially so that the energy is confined near the interface and will not propagate into the material.

Similar to metal-SPP, the surface waves guided by multilayers should also be TM polarized. *H*_y_, *E*_x_, *E*_z_ can be used to describe the electromagnetic field in the space. For z > 0, the field components are:

4 $$\left\{ {\begin{array}{l} {H_{\text{y}} = H_{1} {\text{e}}^{i\beta x} {\text{e}}^{{ - k_{1} z}} } \\ {E_{\text{x}} = iH_{1} \frac{{k_{1} }}{{\omega \varepsilon_{0} \varepsilon_{1} }}{\text{e}}^{i\beta x} {\text{e}}^{{ - k_{1} z}} } \\ {E_{\text{z}} = - H_{1} \frac{\beta }{{\omega \varepsilon_{0} \varepsilon_{1} }}{\text{e}}^{i\beta x} {\text{e}}^{{ - k_{1} z}} } \\ \end{array} } \right.$$

For *z* < 0, we have:

5 $$\left\{ {\begin{array}{l} {H_{y} = H_{2} {\text{e}}^{i\beta x} {\text{e}}^{{k_{2} z}} } \\ {E_{x} = - iH_{2} \frac{{k_{2} }}{{\omega \varepsilon_{0} \varepsilon_{x} }}{\text{e}}^{i\beta x} {\text{e}}^{{k_{2} z}} } \\ {E_{z} = - H_{2} \frac{\beta }{{\omega \varepsilon_{0} \varepsilon_{z} }}{\text{e}}^{i\beta x} {\text{e}}^{{k_{2} z}} } \\ \end{array} } \right.$$

In Eqs. 4 and 5, *k*_1_ = *i* × *k*_z,f_, *k*_2_ = *i* × *k*_z,m_, where *k*_z,f_ and *k*_z,m_ represent wave vector components that is perpendicular to the interface in two areas (*z* > 0 and *z* < 0), respectively. Replacing *k*_z,f_, *k*_z,m_ by *k*_1_, *k*_2_ makes Eqs. 4 and 5 match the form of the evanescent field in *z* direction. At z = 0, the continuity of *H*_y_ requires *H*_1_ = *H*_2_ while the continuity of *E*_*x*_ requires

6 $$\frac{{k_{2} }}{{k_{1} }} = - \frac{{\varepsilon_{x} }}{{\varepsilon_{1} }}$$

The relationship between each component of the wave vector in both areas is as follows [2, 16]:

7 $$k_{1}^{2} = \beta^{2} - \varepsilon_{1} k_{0}^{2}$$

8 $$\frac{{k_{2}^{2} }}{{\varepsilon_{x} }} = \frac{{\beta^{2} }}{{\varepsilon_{z} }} - k_{0}^{2}$$

Solving Eqs. 6–8 gives the dispersion relation between *ω* and *β*:

9 $$\beta^{2} = \frac{{\varepsilon_{1} \varepsilon_{z} (\varepsilon_{x} - \varepsilon_{1} )}}{{\varepsilon_{x} \varepsilon_{z} - \varepsilon_{1}^{2} }}k_{0}^{2}$$

**Fig. 3:**
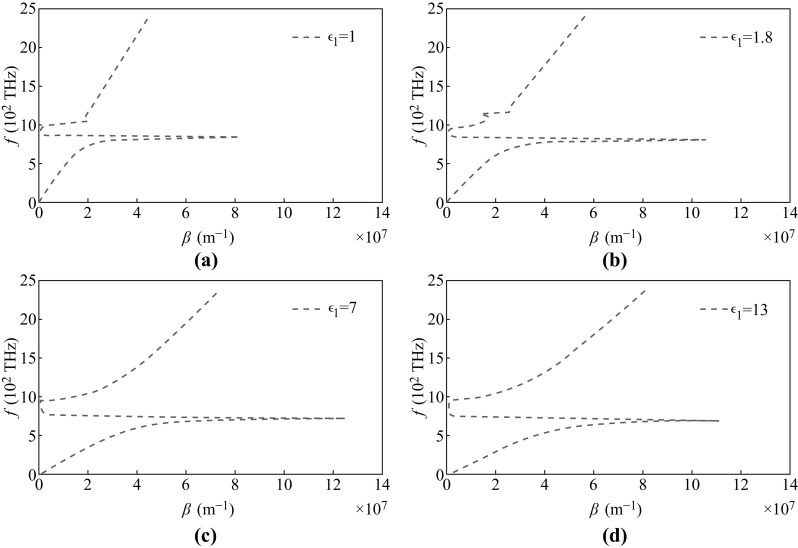
Dispersion curves with different background material (in the area with z > 0). The permittivity *ε*_1_ for *z* > 0 is associated with different kinds of materials [15–17]: **a** air, **b** CH_3_CH_2_OH, **c** TiO_2_ and **d** Si

With Eq. 9, the dispersion relation is obtained and shown in Fig. 3.

## Frequency Range of SPP

Figure 3 shows the dispersion curves of the multilayer meta-material with different background material. Even though the graph shares the similar outline with that of traditional metal-SPP, the dispersion curve for multilayers also has special features that deserve some words. It is well known that traditional metal-SPP has one horizontal asymptote at frequency of $\omega_{\text{sp}} = \frac{{\omega_{\text{p}} }}{{\sqrt {\varepsilon_{\infty } + \varepsilon_{1} } }}$, which also serves as the upper bound for SPP frequency range [12]. Nevertheless, the same rule cannot be applied to SPP on multilayers (multilayer-SPPs) directly, and some discussions are required.

### Frequency Upper Bound

For multilayer-SPPs, the horizontal asymptote appears when and only when *β* goes to infinity. It can be deduced from Eq. 9 that the corresponding condition is

10 $$\varepsilon_{\text{x}} \varepsilon_{\text{z}} - \varepsilon_{1}^{2} = 0$$

In other words, Eq. 10 determines the position of horizontal asymptotes. It also manifests that *ε*_x_ must share the same sign with *ε*_z_ and implies that different *ε*_1_ will result in different curve types.

When *ε*_1_^2^ is larger than *lim*_ω→∞_ *ε*_x_ *ε*_z_, Eq. 10 holds only when both *ε*_x_ and *ε*_z_ are negative, which corresponds to band 2 in Fig. 2a. Therefore, the dispersion curve has only one horizontal asymptote, just like metal-SPP, and the dispersion curve under such circumstance resembles metal-SPP very closely (see Fig. 3c, d).

For smaller *ε*_1_, points within band 2 and band 4 may both satisfy Eq. 10. It may result in two horizontal asymptotes in the dispersion curve and make the curve a little bit different from that of metal-SPP at higher frequency, which is illustrated by Fig. 3a, b.

Similar to *ω*_sp_ for metal, the position of the lowest horizontal asymptote marks the upper bound for SPP (the reason will be discussed in the next section). To distinguish the upper bound of multilayer-SPPs and that of metal-SPP (i.e., *ω*_sp_), the upper bound frequency is denoted as *ω*_upper_. It is easy to verify that multilayer-SPPs can be excited at any frequency lower than *ω*_upper_ in band 2.

### Frequency Lower Bound

**Fig. 4:**
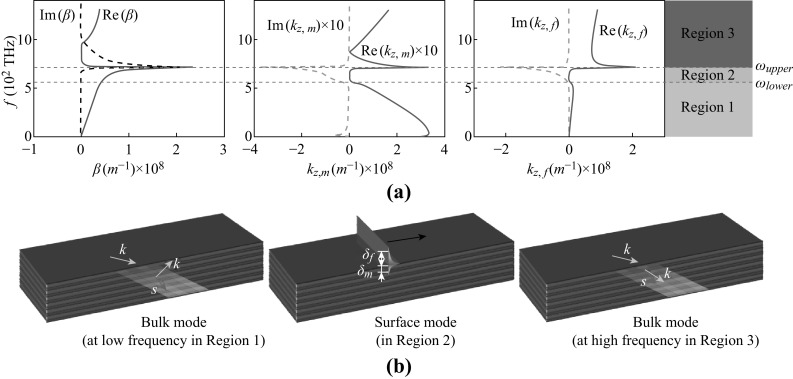
Each component of wave vector. Corresponding to Fig. 3d, the background material is assumed as *ε*_1_ = 13 [18]. **a** The components of *β* and *k* in both multilayers and the free space. **b** The schematic diagram of electromagnetic field in different frequency ranges. Both the left and right figures represent the bulk mode of electro-magnetic field at low frequency (in region 1) and at high frequency (in region 3), respectively, where the wave vectors *k* and the Poynting vectors ***S*** differ in two regions due to different permittivities. The middle figure stands for the surface wave and the depth skins

The dispersion curve above is obtained based on the assumption that the electromagnetic field decays exponentially in *z* direction. Although the dispersion curve shown in Fig. 4a left is similar to that of conventional metal-SPP, not all the mode with frequency below ω_upper_ is SPP here. To obtain the multilayer-SPPs, it is necessary to make sure that both *k*_z,m_ and *k*_z,f_ are imaginary. According to Fig. 4, the frequency is cut into three regions by two cut-off points, *ω*_lower_ and *ω*_upper_. *ω*_upper_ has been defined in Sect. 4.1 as the frequency of the lowest horizontal asymptote in the dispersion curve, while *ω*_lower_, lower than *ω*_upper_, is the frequency where both *k*_z,m_ and *k*_z,f_ convert from real to imaginary. This section will later prove that the real-to-imaginary turning points for *k*_z,m_ and *k*_z,f_ are the same.

As shown in Fig. 4a, in region 1, the real part of *k*_z,m_ in multilayers exists while the imaginary part is negligible, which indicates that the electromagnetic wave can propagate into the material with little damping. Region 1 ends at the frequency *ω*_lower_ and region 2 begins here. In region 2, *k*_z,m_ has relatively large imaginary part and negligible real part. It means that the field is evanescent in this region. When the frequency is higher than *ω*_upper_ (in region 3), the real part of *k*_*z,m*_ emerges and the imaginary part disappears again. The right figure in Fig. 4a displays the wave vector component *k*_z,f_ in the free space. Similarly, the wave can propagate into the free space in region 1 and 3, while it is bounded to the interface in region 2.

Now it is the time to prove that the real-to-imaginary turning points *ω*_lower_ for both *k*_z,m_ and *k*_z,f_ are the same. Suppose that the real-to-imaginary turning points for *k*_z,m_ is *ω*_lower_ while that for *k*_z,f_ is *ω*_lower,2_.

In multilayers (*z* < 0), Eq. 8 can be rewritten in the following form:

11 $$\frac{{\beta^{2} }}{{\varepsilon_{\text{z}} }} + \frac{{k_{\text{z,m}}^{2} }}{{\varepsilon_{\text{x}} }} = k_{0}^{2}$$

It is known that *ω*_upper_ is in band 2 and the concerned turning point (*ω*_lower,1_ and *ω*_lower,2_) is below *ω*_upper_. The discussion in previous sections implies that this turning point is within band 1 (*ε*_x_ < 0, *ε*_z_ > 0). The multilayers can be regarded as the hyperbolic material in this band and Eq. 11 turns to be

12 $$\frac{{\beta^{2} }}{{\left| {\varepsilon_{z} } \right|}} - \frac{{k_{\text{z,m}}^{2} }}{{\left| {\varepsilon_{x} } \right|}} = k_{0}^{2}$$

For evanescent field in multilayers, *k*_z,m_ should be imaginary ($k_{{{\text{z}},{\text{m}}}}^{ 2} < 0$) and we obtain

$$\frac{{\beta^{2} }}{{\left| {\varepsilon_{\text{z}} } \right|}} - k_{0}^{2} = \frac{{k_{\text{z,m}}^{2} }}{{\left| {\varepsilon_{\text{x}} } \right|}} < 0 \Rightarrow \beta^{2} < \varepsilon_{\text{z}} k_{0}^{2}$$

Applying Eq. 9, we have

13 $$\frac{{\varepsilon_{1} \varepsilon_{\text{z}} (\varepsilon_{\text{x}} - \varepsilon_{1} )}}{{\varepsilon_{\text{x}} \varepsilon_{\text{z}} - \varepsilon_{1}^{2} }} < \varepsilon_{\text{z}} \Rightarrow \varepsilon_{1} < \varepsilon_{\text{z}}$$

Thus, to have the evanescent field in multilayers, condition (Eq. 13) is required. According to the definition that *ω*_lower,1_ is the real-to-imaginary turning points for *k*_z,m_, *ω*_lower,1_ should correspond to the critical point of (Eq. 13). In other words, *ω*_lower,1_ marks the frequency at which *ε*_1_ = *ε*_z._

In the dielectrics above multilayers (z > 0), we rewrite (Eq. 7) as

14 $$\beta^{2} + k_{\text{z,f}}^{2} = \varepsilon_{1} k_{0}^{2}$$

Similarly, the evanescent field requires *k*_z,f_ to be imaginary (*k*_z,f_^2^ < 0) so that

15 $$\beta^{2} - \varepsilon_{1} k_{0}^{2} = k_{\text{z,f}}^{2} < 0 \Rightarrow \beta^{2} > \varepsilon_{1} k_{0}^{2} \Rightarrow \frac{{\varepsilon_{1} \varepsilon_{\text{z}} (\varepsilon_{\text{x}} - \varepsilon_{1} )}}{{\varepsilon_{\text{x}} \varepsilon_{\text{z}} - \varepsilon_{1}^{2} }} > \varepsilon_{1} \Rightarrow \varepsilon_{\text{z}} > \varepsilon_{1}$$

*ω*_lower,2_, the real-to-imaginary turning points for *k*_z,f_, corresponds to the critical condition for (Eq. 15), i.e., *ε*_1_ = *ε*_z_, which is the same as the condition for *ω*_lower,1_. Hence, the lower cut-off point can be uniquely denoted as *ω*_lower_.

Now, we assert that the SPP appears only within region 2, which spans from *ω*_lower_ to *ω*_upper_. The corresponding condition is

16 $$\begin{aligned} \varepsilon_{\text{z}} > \varepsilon_{ 1} \left( {\text{in band 1}} \right) {\text{ }{\rm or}} \hfill \\ \varepsilon_{\text{x}} \varepsilon_{\text{z}} - \varepsilon_{ 1}^{ 2} < 0 \, \left( {\text{in band 2}} \right) \hfill \\ \end{aligned}$$

In this range, the SPP can be excited by evanescent field (using prisms [17], electrons [19–21], etc.). When the wave vector in the propagation direction is matched, the multilayer-SPPs will be excited and propagate stably.

### Frequency Range

**Fig. 5:**
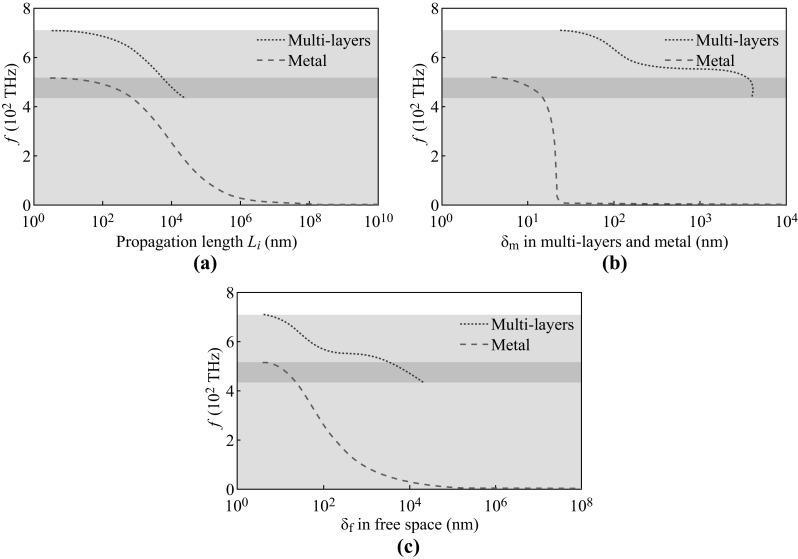
**a** Propagation length, **b** skin depth in multilayers, and **c** skin depth in free space of metal-SPP and multilayers-SPP. The blue and red colors are to mark the frequency range of multilayer-SPPs and metal-SPP, respectively

Knowing the lower and upper bound of multilayer-SPPs, its frequency is completely determined. Figure 5 displays the frequency range of multilayer-SPPs (blue part), together with that of metal-SPP (red part). The metal-SPP frequency range spans from 0 to 517 THz, while the multilayer-SPPs spans from 434 to 710 THz. It can be clearly observed that multilayer-SPPs has higher and narrower frequency range than metal. As we know, the surface plasmon results from the oscillation of free electrons at the metal surface. Intuitively, the decrease of the number of free electrons would lead to the reduction of frequency range since the negativity of *ε*_m_ is correlated to the density of free electrons positively. In general cases, the metal with lower free electron density indeed displays a lower *ω*_sp_, which marks the frequency range upper bound. Here, the multilayers with less free electron density by replacing some parts of the metal with dielectric layers should lead to the decreased *ω*_sp_. However, the structure of multilayers contradicts with this intuition. The computation implies that this structure has the ability to extend the SPP to some higher frequency where even metal-SPP cannot exist.

**Fig. 6:**
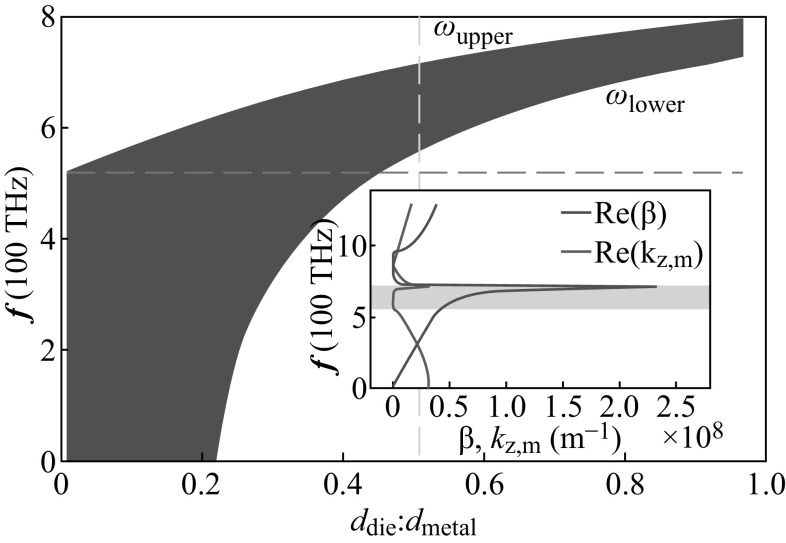
The blue area determined by *ω*_upper_ and *ω*_lower_ represents the frequency range of multilayers-SPP. The *red dashed line* marks *ω*_sp_ for pure metal (silver). Corresponding to the *orange dashed line*, the inset reveals the dispersion curve of multilayers-SPP as *d*_metal_:*d*_die_ = 0.5. (Color figure online)

Inspired by this counterintuitive conclusion, we further increase the ratio of dielectric layers in the multilayers structure. Figure 6 shows the SPP frequency range as the depth ratio *d*_die_:*d*_metal_ goes from 0.01 to 1. As we have assumed that *d*_metal_ ≥ *d*_die_, the ratio *d*_die_:*d*_metal_ will not be greater than 1. From the figure, it is clear that the upper bound of frequency range has been elevated to nearly 800 THz when *d*_die_:*d*_metal_ ≈ 1, while metal-SPP can only reach 500 ~ 550 THz under the same condition. This improvement works when the background material has high permittivity. In our case, we use Si (whose permittivity is around 13) to fill the area *z* > 0. The figure indicates that the thicker the dielectric layers in multilayers are, the higher multilayer-SPPs frequency range can reach. It also contradicts with the intuition.

## Propagation Length and Skin Depth

Obtaining the wave vectors, the characteristic length of multilayer-SPPs can be computed and analyzed. The propagation length and skin depth are expressed as [22, 23],

17 $$L_{i} = \frac{1}{{2{\text{Im}}(\beta )}}$$

18 $$\delta_{\text{m}} = \frac{1}{{{\text{Im}}(k_{\text{z,m}} )}}$$

19 $$\delta_{\text{f}} = \frac{1}{{{\text{Im}}(k_{\text{z,f}} )}}$$

Here, *L*_*i*_ stands for the propagation length in *x* direction, and *δ*_m_, *δ*_f_ stand for the skin depths in the material (metal or multilayers) and free space, respectively. Setting *ε*_1_ = 13, Fig. 5 shows the above feature length of both metal-SPP and multilayer-SPPs. It is clear that the propagation length of multilayer-SPPs is around 5 times longer than that of metal-SPP at the same frequency. Thus, the propagation loss of multilayer-SPPs is much lower than that of metal-SPP. This is because more field of multilayer-SPPs expands into the dielectric area (*z* > 0) (Fig. 5c) and meanwhile exists in the dielectric parts of multilayers structure (*z* < 0) (Fig. 5b). Thus, in some circumstances, using the multilayers structure to substitute original metal material would result in longer propagation length. And by adjusting the ratio of metal and dielectric in multilayers, the properties of SPP mode could be tuned.

## Discussion

For traditional metal-SPP, to fulfill the electromagnetic boundary condition, the absolute value of Re(*ε*_m_) should be larger than dielectric permittivity *ε*_1_, and the electric field component *E*_x_ should not be larger than *E*_z_. Otherwise, the SPP mode would be cutoff. We assume that *E*_*z*_ smaller than *E*_x_ is a criterion for judging the existence of SPP mode, although it is not rigorous for multilayers structure.

From the perspective of effective medium, multilayers present the property of anisotropic material under certain circumstances, as shown in Sect. 2. Further, Fig. 2 indicates that multilayers could be regarded as anisotropic metal near the upper bound of its SPP frequency range (in band 2). Within band 2 of Fig. 2a, the Re(*ε*_z_) is far from zero, while Re(*ε*_x_) is much closer to zero, compared with the Re(*ε*_m_) of pure metal.

Due to the much larger absolute value of Re(*ε*_z_) than that of Re(*ε*_x_), considering the continuity of electric field *E*_x_ along x and electric displacement field *ε*_i_·*E*_z_ (*i* = 1 or z) along z at *z* = 0, the electric field component *E*_z_ could be kept smaller than *E*_x_ even though the dielectric permittivity *ε*_1_ is increased a lot. Thus, the SPP could be supported by the multilayers with high *ε*_1_ according to the non-rigorous criterion mentioned above.

Since the extension of SPP frequency is due to the abnormal effective permittivity of multilayer structure along x and z direction. The effective permittivity results from the interaction of electromagnetic field with the free electrons in metal layers and the SPP mode coupling and resonant in multilayer metal-dielectric structure. Therefore, the extension of SPP frequency range is ascribed to the SPP mode coupling and resonant in the multilayers with anisotropic permittivity.

By the way, the multilayer-SPPs could also be excited by evanescent electromagnetic field generated by prism, grating or waveguide. The extended frequency range, lower propagation loss, and tunable properties by changing the metal-dielectric ratio might be useful for biosensor, integrated circuits, active SPP devices.

## Conclusions

The SPP guided by metal/dielectric multilayers meta-material was studied theoretically. Regarding the meta-material as an anisotropic material by the effective medium theory, the dispersion relation of the SPP was derived. It is revealed that SPP can be supported by the meta-material at the frequency where conventional metal-SPP cannot exist when the permittivity of dielectric on metal is high. Besides the difference of high frequency cutoff point compared with metal-SPP, it was found that there exists a low cut-off frequency, below which SPP could not be supported by the meta-material. The calculation results also revealed that the multilayer-SPPs has larger propagation length and skin depth compared with metal-SPP. Therefore, at some specific frequency, multilayers could be more favorable than metal in transmitting signals.

These amazing properties provide the possibility to make use of SPP mode for some potential applications, which are impossible for metal-SPP, such as optical interconnection and active SPP devices, where Si and active III-V material (GaAs) with high permittivity might result in the cutoff of SPP mode or extremely large loss at certain frequency. Besides, the characteristics of the multilayer-SPPs are easy to be manipulated by adjusting the structure parameters and the active materials can also be introduced into the meta-material, which might bring more interesting features to future devices.
